# Synergistic Regulation of Microstructure and Properties in Al-Zr Alloys via Sc Addition and Ultrasonic Treatment

**DOI:** 10.3390/ma19091792

**Published:** 2026-04-28

**Authors:** Jincheng Sun, Xun Wang, Yang An, Chao Ying, Yuanzheng Yang, Yuliang Zhao

**Affiliations:** 1 Postdoctoral Research Station of Materials Science and Engineering, Guangdong University of Technology, Guangzhou 510006, China; sun.jincheng.q8@alumni.tohoku.ac.jp; 2 Guangzhou Cable Co., Ltd., Guangzhou 511468, China; 3 School of Mechanical Engineering, Dongguan University of Technology, Dongguan 523808, China

**Keywords:** Al-Zr alloy, Sc addition, ultrasonic treatment, grain refinement, electrical conductivity, mechanical properties

## Abstract

Heat-resistant Al–Zr conductors are limited by the strength–conductivity trade-off and by long aging schedules required to stabilize Al_3_Zr-based precipitates. This work investigates the combined effect of scandium addition (0–0.30 wt.%) and ultrasonic treatment (UST) during melt processing on Al–0.3Zr–xSc alloys. UST was applied at 710 °C before casting; phase-equilibrium analysis and quantitative measurements of intermetallic distribution, grain size, electrical conductivity, and tensile properties were performed before and after 25 h aging. Grain refinement shows a clear Sc-dependent threshold: UST refines the Sc-free alloy to ~177 μm, whereas 0.05 wt.% Sc causes abnormal coarsening (~396 μm). Increasing Sc to 0.10–0.20 wt.% produces pronounced refinement (~110 to ~82 μm), and the refined grain structures are retained after aging. At 0.20 wt.% Sc, the aged alloy achieves >100 MPa tensile strength while recovering approximately 58% IACS (International Annealed Copper Standard). Overall, the results reveal a composition-dependent synergy between Sc microalloying and UST that enables microstructure control and an improved strength–conductivity balance, with potential to contribute to more efficient processing strategies for heat-resistant aluminum conductors.

## 1. Introduction

Aluminum (Al) and its alloys have long been recognized as indispensable materials for electrical engineering applications, particularly in power transmission and distribution systems, owing to their low density, high specific strength, excellent electrical conductivity, and satisfactory corrosion resistance [1]. Compared with traditional copper conductors, aluminum-based conductors offer significant advantages in terms of weight reduction and cost efficiency, which becomes increasingly critical under the current global trend of rising copper prices and growing demand for large-scale power infrastructure [2]. As power transmission capacity continues to increase, modern conductors are required to carry higher current densities over extended periods, inevitably resulting in elevated operating temperatures. Under such conditions, conventional aluminum conductors suffer from severe strength degradation, creep deformation, and microstructural instability, which significantly limit their long-term reliability and service life [3].

To address these challenges, heat-resistant aluminum alloys have been extensively developed and applied in electrical conductors operating at elevated temperatures. Among them, Al-Zr alloys have attracted particular attention as representative medium-strength, heat-resistant aluminum conductors [4]. The excellent thermal stability of Al-Zr alloys primarily originates from the precipitation of Al_3_Zr intermetallic compounds and the extremely low diffusion coefficient of zirconium (Zr) in the aluminum matrix [5]. The slow diffusion of Zr effectively suppresses the coarsening of Al_3_Zr precipitates during long-term exposure to high temperatures, thereby maintaining the mechanical strength and creep resistance of the alloy [3]. As a result, Al-Zr alloys have been widely employed in heat-resistant conductor wires where both electrical conductivity and thermal stability are required.

Despite these advantages, the performance of Al-Zr alloys is still constrained by the intrinsic trade-off between mechanical strength and electrical conductivity [2]. On the one hand, increasing the amount of alloying elements and precipitates can enhance strength and thermal stability; on the other hand, excessive solute atoms and intermetallic phases inevitably increase electron scattering, leading to a reduction in electrical conductivity according to Matthiessen-type descriptions [6]. In practical applications, achieving a favorable balance between strength and conductivity remains a critical challenge, especially for conductors required to operate at temperatures exceeding 120 °C for prolonged durations [7].

Rare earth (RE) microalloying has been widely explored as an effective strategy to further enhance the mechanical performance and thermal stability of Al-Zr alloys [8,9]. Among various RE elements, scandium (Sc) is recognized as one of the most potent strengthening elements for aluminum alloys [10]. The addition of Sc leads to the precipitation of coherent Al_3_Sc particles, which provide strong precipitation strengthening due to their excellent lattice coherence with the aluminum matrix [11]. Moreover, Al_3_Sc particles can act as heterogeneous nucleation sites for Al_3_Zr, promoting the formation of thermally stable Al_3_(Zr, Sc) composite precipitates [5,12]. These composite precipitates exhibit superior resistance to coarsening at elevated temperatures compared with Al_3_Zr alone, thereby offering enhanced thermal stability and creep resistance [3]. From a microstructural design perspective, this synergistic precipitation behavior makes Sc an attractive microalloying element for heat-resistant conductor alloys, particularly in applications where long-term thermal exposure is unavoidable.

As a consequence, Al–Zr–Sc alloys have been extensively investigated for high-temperature conductor applications, and numerous studies have demonstrated that Sc microalloying can significantly enhance tensile strength and high-temperature performance [13,14,15,16,17]. Nevertheless, these benefits are inherently accompanied by several critical limitations. Notably, these limitations are not purely compositional in nature, but are closely coupled with processing routes and precipitation kinetics, which further complicates the optimization of Al–Zr–Sc conductor alloys. From an industrial perspective, the high cost and limited availability of scandium severely constrain its large-scale application [7]. From a microstructural standpoint, excessive Sc addition promotes the formation of coarse intermetallic particles, which in turn leads to a deterioration in ductility [18,19]. More fundamentally, achieving high electrical conductivity (typically ≥60% IACS) in Al-Zr-Sc alloy conductor rods relies on prolonged heat treatment to ensure sufficient precipitation of Sc- and Zr-containing phases, with aging durations frequently extending to several tens or even hundreds of hours [20,21,22].

When translated into large-scale industrial manufacturing, such processing requirements inevitably result in high energy consumption, extended production cycles, and reduced industrial efficiency [23]. This issue becomes particularly pronounced for conductor alloys, where production throughput and energy efficiency are critical factors governing large-scale deployment in power transmission infrastructure. In industrial practice, the manufacture of heat-resistant aluminum conductor rods is usually conducted in large-scale melting furnaces, followed by casting and long-duration heat treatment. For example, an industrial-scale furnace requires several hours for melting and casting, and the produced rods often undergo aging treatments lasting tens to hundreds of hours to ensure adequate precipitation strengthening and thermal stability [4]. Such processing routes represent a major bottleneck for the large-scale application of high-performance Al-Zr-Sc conductor alloys. Therefore, developing more efficient processing strategies to shorten heat treatment time while maintaining a favorable balance between mechanical properties and electrical conductivity is of great technological and economic significance.

In recent years, ultrasonic treatment (UST) of molten metals has emerged as an effective approach to refine solidification microstructures and improve alloy homogeneity [24,25,26,27,28,29]. Ultrasonic irradiation introduces high-frequency mechanical vibrations into the melt, generating complex physical phenomena such as cavitation and acoustic streaming. Cavitation involves the formation, growth, and violent collapse of bubbles, producing localized high pressures and temperatures, shock waves, and micro-jets. Acoustic streaming, arising from the attenuation of ultrasonic energy in the melt, induces steady-state fluid flow, which enhances melt convection and solute transport. The combined effects of cavitation and acoustic streaming can significantly influence nucleation, particle fragmentation, solute distribution, and solidification dynamics.

Extensive studies have demonstrated that ultrasonic treatment can effectively refine grains, reduce porosity, and improve the uniformity of microstructures in aluminum alloys [30]. In the context of Al-Zr alloys, ultrasonic treatment has been shown to strongly affect the behavior of Al_3_Zr intermetallic compounds in the melt. In our previous work on the Al-Zr system, the behavior of Al_3_Zr intermetallic particles under high-amplitude ultrasonic irradiation was systematically investigated [31,32]. It was revealed that the influence of ultrasonic treatment on Al_3_Zr particles is not limited to conventional concepts such as particle fragmentation or enhanced heterogeneous nucleation. Instead, cavitation-induced heat generation and microjet-driven mass transfer were identified as critical mechanisms governing the morphology and growth of Al_3_Zr intermetallic compounds. With increasing ultrasonic vibration amplitude, needle-like Al_3_Zr particles underwent a transition toward thicker and more rounded morphologies, indicating a substantial modification of intermetallic growth behavior in the molten state. These observations suggest that ultrasonic treatment may influence not only solidification-related phenomena, but also the initial state of intermetallic particles that governs subsequent precipitation behavior during heat treatment.

These findings provide important mechanistic insight into how ultrasonic treatment can regulate the initial microstructural state of Al-Zr alloys prior to solidification. By modifying the morphology, size, and spatial distribution of primary Al_3_Zr intermetallic compounds, ultrasonic treatment can effectively reduce microstructural heterogeneity inherited from casting and establish more favorable initial conditions for subsequent heat treatment. However, while the effects of ultrasonic treatment on Al-Zr alloys have been clarified to a considerable extent, its interaction with Sc microalloying in Al-Zr-Sc systems remains insufficiently understood.

In particular, the combined effect of ultrasonic treatment and Sc addition on grain refinement, precipitation behavior, and the balance between electrical conductivity and mechanical properties has not yet been systematically investigated. Existing studies often focus on either Sc microalloying or ultrasonic treatment as an independent variable, whereas their synergistic interaction, especially under different Sc content regimes, has received limited attention. Moreover, potential non-monotonic or threshold-dependent behaviors associated with Sc addition under ultrasonic treatment conditions have rarely been reported, despite their critical importance for composition optimization.

Therefore, the present study aims to systematically investigate the synergistic effects of Sc addition and ultrasonic treatment on the microstructure and properties of Al-Zr alloys. Al-0.3Zr-xSc alloys with varying Sc contents were prepared under ultrasonic treatment conditions, and their phase equilibrium, microstructural evolution, grain size, electrical conductivity, and tensile properties were comprehensively characterized. Particular emphasis was placed on identifying composition-dependent transitions in dominant microstructural control mechanisms and evaluating their implications for heat treatment efficiency. The results of this study are expected to provide both mechanistic understanding and practical guidance for optimizing Al-Zr-Sc alloy processing, with the ultimate goal of shortening heat treatment time and improving the industrial viability of high-performance heat-resistant aluminum conductor alloys.

## 2. Experimental Procedures

### 2.1. Ultrasonic Treatment, Melting and Casting

To determine suitable ultrasonic processing parameters, the Al–Zr–0.1Sc alloy was selected for preliminary ultrasonic treatment experiments. Based on the phase equilibrium analysis, the ultrasonic treatment temperatures were set at 680 °C, 710 °C, and 740 °C. Considering a melt mass of 2 kg, the ultrasonic treatment duration was fixed at 60 s (corresponding to 30 s per kilogram of melt). An ultrasonic frequency of 40 kHz, which is commonly adopted to ensure stable cavitation in aluminum melts, was employed throughout the experiments.

The ultrasonic power density (PD) was estimated for reference using the relation (1) [33]:

(1) $$\mathrm{PD}\;=\;\frac{P_{\mathrm{acoustic}}}{P_{\mathrm{rad}}}$$

where the effective acoustic power *P*_acoustic_ was approximately 500 W and the acting area of the horn tip *P*_rad_ was about 7 cm^2^, resulting in an estimated power density of approximately 70 W·cm^−2^.

**Figure 1:**
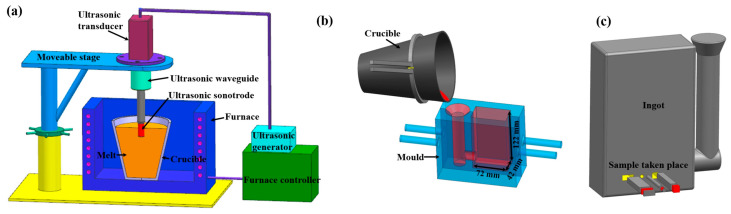
Schematic illustration of the ultrasonic treatment and casting process: (**a**) ultrasonic treatment setup; (**b**) steel mold; and (**c**) as-cast ingot with sampling positions [34].

The ultrasonic treatment system, schematically illustrated in Figure 1a, consisted of an ultrasonic generator and a titanium alloy horn. Industrial-purity aluminum (99.8 wt.%) and an Al–Sc master alloy were used as raw materials and weighed according to the target compositions. Melting was carried out in a pit-type resistance furnace using a graphite crucible at 800–850 °C, and the temperature was maintained until complete melting was achieved. Degassing and melt refining were conducted using hexachloroethane (C_2_Cl_6_) at approximately 760 °C, followed by slag removal and a holding period of 10 min.

When the melt temperature decreased to the preset ultrasonic treatment temperature, the ultrasonic horn was immersed into the melt, and ultrasonic irradiation was applied for the predetermined duration. After ultrasonic treatment, the horn was withdrawn, and the melt was immediately poured into a steel mold, as shown in Figure 1b. The as-cast ingot and the corresponding sampling locations are presented in Figure 1c.

### 2.2. Thermodynamic Calculations

Thermodynamic phase-equilibrium calculations were performed using Thermo-Calc software (Thermo-Calc 2025A, Stockholm, Sweden). The TCAL5 aluminum alloy database (version 5) was employed for the CALPHAD simulations to generate equilibrium phase diagrams and evaluate phase stability under the relevant processing conditions. All calculations were conducted under equilibrium assumptions.

### 2.3. Chemical Composition Analysis

The nominal compositions and measured elemental contents of the alloys are listed in Table 1. Chemical composition analysis was performed using inductively coupled plasma optical emission spectroscopy (ICP-OES, Agilent 720, Agilent Technologies, Santa Clara, CA, USA). Each sample was analyzed at least three times, and the average values were adopted for subsequent analysis.

### 2.4. Microstructural Characterization

#### 2.4.1. Sample Preparation

Samples for microstructural characterization were sectioned from representative regions of the as-cast ingots. The typical dimensions of the samples were approximately 10 mm × 10 mm × 5 mm. Standard metallographic procedures, including grinding and polishing, were employed to obtain flat and deformation-free surfaces suitable for microstructural observation.

#### 2.4.2. SEM Observation and EDS Analysis

Microstructural observations were conducted using a ZEISS Sigma 500 scanning electron microscope (SEM, ZEISS, Oberkochen, Germany) equipped with an energy-dispersive X-ray spectroscopy (EDS) system. Prior to observation, the samples were sputter-coated with a thin gold layer (approximately 5–10 nm) to minimize surface charging.

SEM examinations were performed under high-vacuum conditions (better than 1 × 10^−5^ Pa). Appropriate accelerating voltage, working distance, and magnification were selected to observe grain morphology, intermetallic phases, and precipitate distributions. Based on SEM images, representative regions such as intermetallic particles and grain boundary areas were selected for EDS point or area analysis. During EDS measurements, the accelerating voltage was set to 15–20 kV, and the acquisition time ranged from 10 to 30 s to ensure sufficient characteristic X-ray signal collection.

#### 2.4.3. Grain Size Measurement

Grain structures were revealed using the anodic oxidation film method, followed by quantitative grain size analysis using the linear intercept method. During anodic oxidation, the polished sample served as the anode and a stainless-steel plate as the cathode, both immersed in a boric acid–methanol electrolyte. A direct current voltage of 10–20 V and a current density of 0.5–1 A·dm^−2^ were applied for 3–5 min. After oxidation, the samples were rinsed with deionized water and air-dried.

Grain size measurements were conducted using an optical microscope (TMR2000 polarized light microscope, Qingdao, China) at a magnification of 100×. At least 10 representative fields of view were selected for each sample. In each field, three to five test lines of equal length (typically 1000 μm) were drawn, and the number of intersections between the test lines and grain boundaries was counted. According to ASTM E112 [35], the grain size number *G* was calculated as Formula (2):

(2) $$G\;=\;-6.643856\log(\bar{l})-3.288$$

where $\bar{l}$ is the average grain intercept length. The final grain size was obtained by averaging the results from all measured fields.

### 2.5. Tensile Testing

Tensile strength and elongation were measured using an EM6.204-L universal testing machine (TSMI, EM6.304-L, Taiwan SUGA Test Instruments Co., Ltd., Taiwan, China) at room temperature. Tensile specimens were prepared in accordance with the ASTM E8/E8M standard for metallic materials [36]. All tests were conducted under displacement-controlled loading at a constant strain rate specified by the standard. For each condition, at least three specimens were tested to ensure reproducibility, and the average values were reported as the final results.

### 2.6. Heat Treatment and Electrical Conductivity Measurement

Heat treatment was conducted in a controlled air-circulating furnace (KSL-1100X mini box Furnace, Hefei Kejing Materials Technology Co., Ltd., Hefei, China) at 425 °C under isothermal aging conditions to promote the precipitation of Zr- and Sc-containing phases. Electrical conductivity measurements were performed at fixed time intervals of 5 h to monitor the evolution of conductivity during aging. The total aging duration was limited to 25 h, after which no significant change in conductivity was observed, indicating that a near-steady-state condition had been reached.

Electrical conductivity was determined using an FD102 digital portable eddy-current conductivity meter (Xiamen First Company, Xiamen, China). The instrument was calibrated prior to testing, and sample surfaces were cleaned to minimize measurement variability. Measurements were performed with the probe placed perpendicular to the sample surface. At least three measurements were taken at different locations on each specimen, and the average value was used for analysis.

## 3. Results

### 3.1. Phase Equilibrium Analysis of Al-0.3Zr-xSc Alloys at Ultrasonic Treatment Temperature

**Figure 2:**
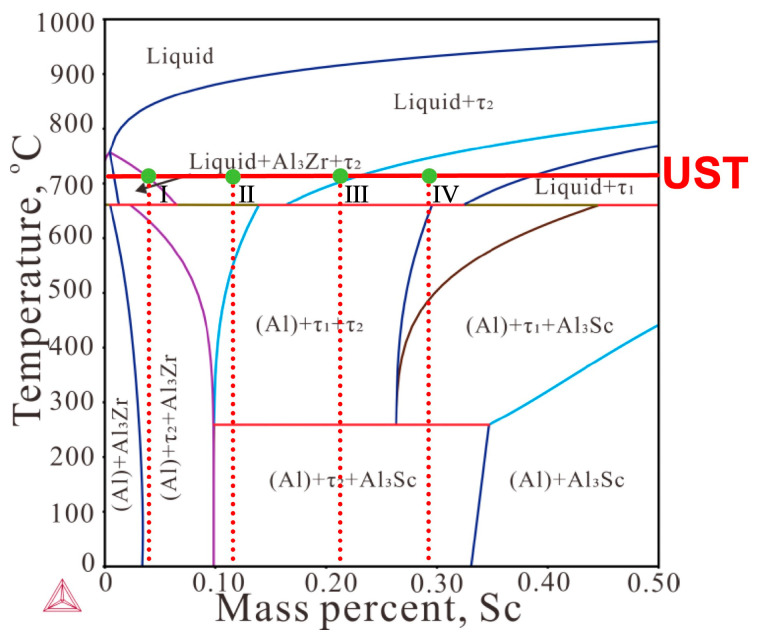
Calculated Al-Zr-Sc phase diagram. The dashed vertical lines indicate the studied compositions (Al-0.3Zr-xSc), and the solid horizontal line marks the UST temperature.

The phase evolution and equilibrium phase assemblages of Al-0.3Zr-xSc alloys (x = 0–0.30 wt.%) at 710 °C, corresponding to the isothermal temperature for ultrasonic treatment prior to casting, were analyzed based on the calculated Al-Zr-Sc phase diagram, as shown in Figure 2. The red dashed lines indicate the phase boundaries at different Sc contents, while the solid red line represents the ultrasonic treatment temperature.

For the Sc-free alloy, the system belongs to the Al-Zr binary subsystem at a fixed Zr content and is located in the liquid (L) + Al_3_Zr two-phase field at 710 °C. Under this condition, ultrasonic treatment was applied to a molten aluminum matrix coexisting with solid Al_3_Zr intermetallic particles. When 0.05 wt.% Sc was added, the phase equilibrium shifted into the L + Al_3_Zr + τ_2_ three-phase field, indicating the formation of a ternary intermetallic phase (τ_2_) involving Al, Sc, and Zr. In this case, ultrasonic treatment interacted simultaneously with the liquid matrix and two types of solid intermetallic particles.

With further increasing Sc content to 0.10 wt.%, the equilibrium phase assemblage transformed into the L + τ_2_ two-phase field, suggesting that the Al_3_Zr phase was completely dissolved due to the increased Sc addition. Ultrasonic treatment under this condition primarily interacted with τ_2_ particles dispersed in the melt. At a Sc content of 0.20 wt.%, the system remained within the L + τ_2_ phase region at 710 °C, although the equilibrium state approached the boundary of the L + τ_1_ + τ_2_ three-phase field. When the Sc content reached 0.30 wt.%, the alloy entered the L + τ_1_ + τ_2_ three-phase field, where τ_1_ represents another ternary intermetallic phase in the Al-Sc-Zr system.

Based on thermodynamic predictions, the equilibrium phases at room temperature were determined to be (Al) + Al_3_Zr for the Sc-free alloy, (Al) + Al_3_Zr + τ_2_ for the alloy containing 0.05 wt.% Sc, and (Al) + τ_1_ + τ_2_ for alloys with Sc contents ranging from 0.10 to 0.30 wt.%. These results provide a thermodynamic basis for understanding the differences in microstructural evolution observed in alloys with varying Sc contents under ultrasonic treatment conditions.

### 3.2. As-Cast Microstructure and Elemental Distribution

**Figure 3:**
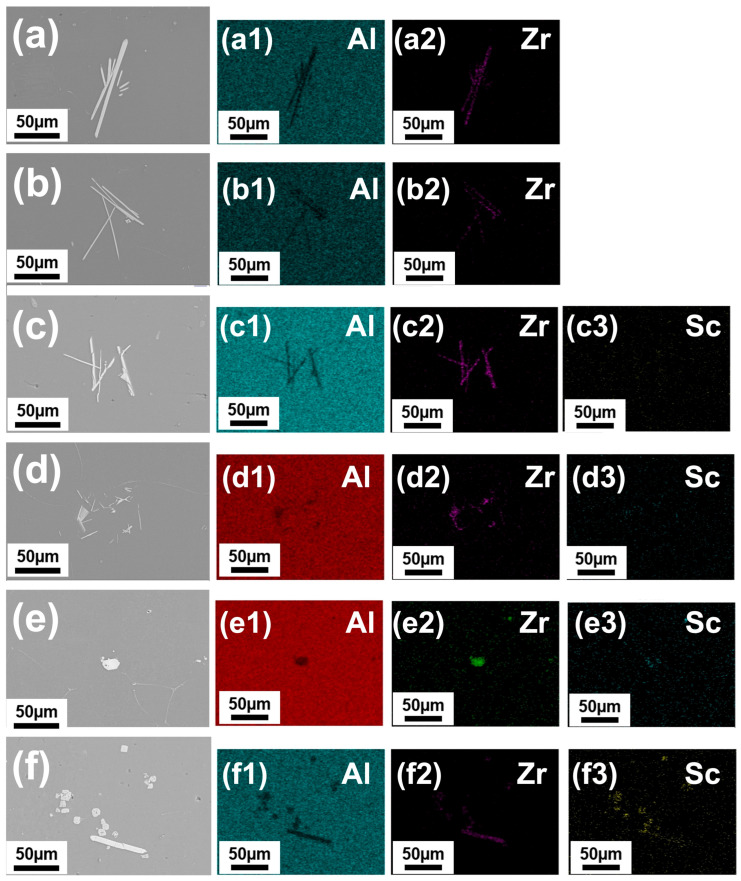
High-magnification SEM micrographs highlighting intermetallic morphology and corresponding EDS elemental mappings of Al–0.3Zr–xSc alloys: (**a**) Al–0.3Zr alloy without ultrasonic treatment; (**b**) Al–0.3Zr alloy with ultrasonic treatment; (**c**–**f**) Al–0.3Zr–xSc alloys with x = 0.05, 0.10, 0.20 and 0.30 wt.%, respectively. (**a1**) Al map and (**a2**) Zr map of the Al–0.3Zr alloy without ultrasonic treatment; (**b1**) Al map and (**b2**) Zr map of the Al–0.3Zr alloy with ultrasonic treatment; (**c1**–**f1**), (**c2**–**f2**), and (**c3**–**f3**) correspond to the EDS elemental maps of Al, Zr, and Sc, respectively.

**Figure 4:**
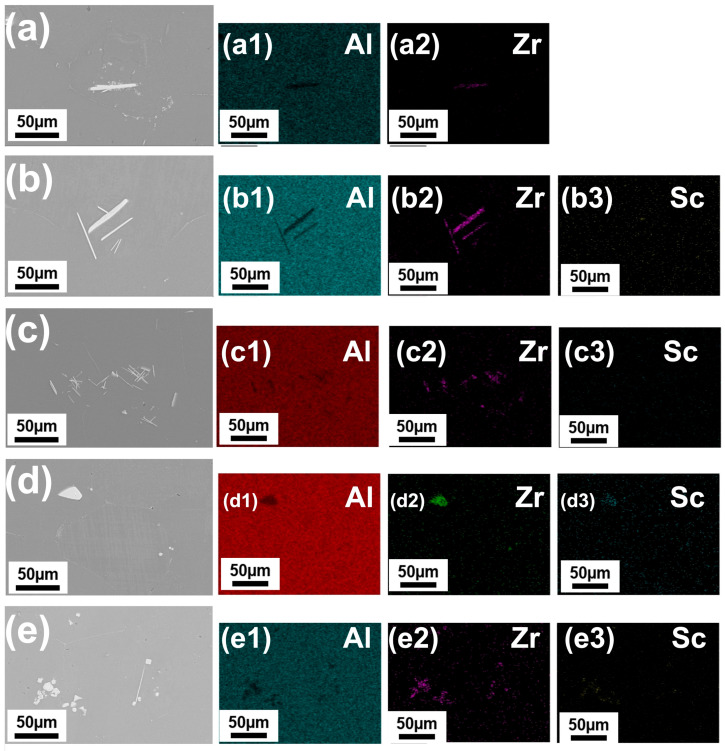
High-magnification SEM micrographs highlighting intermetallic morphology and corresponding EDS elemental mappings of Al–0.3Zr–xSc alloys after 25 h heat treatment (x = 0–0.30 wt.%). (**a**) Al–0.3Zr alloy without ultrasonic treatment; (**b**–**e**) Al–0.3Zr–xSc alloys with x = 0.05, 0.10, 0.20 and 0.30 wt.%, respectively. (**a1**) Al map and (**a2**) Zr map of the Al–0.3Zr alloy without ultrasonic treatment; (**b1**–**e1**), (**b2**–**e2**), and (**b3**–**e3**) correspond to the EDS elemental maps of Al, Zr, and Sc, respectively.

In order to analyze the morphology and compositional evolution of the Al–Zr and Al–Zr–Sc intermetallic compounds, high-magnification SEM images were employed, as shown in Figure 3 and Figure 4. Figure 3 presents SEM micrographs and corresponding EDS elemental mappings of the as-cast Al-0.3Zr-xSc alloys. For the Al-0.3Zr alloy without ultrasonic treatment (Figure 3a), coarse second-phase particles with irregular morphologies were observed, and Zr was locally enriched in these regions, indicating the formation of Al_3_Zr intermetallics with an inhomogeneous spatial distribution. After ultrasonic treatment (Figure 3b), the size of these intermetallic particles was noticeably reduced, and their distribution became more uniform throughout the aluminum matrix.

With the introduction of Sc, the microstructural characteristics changed significantly. In the Al-0.3Zr-0.05Sc alloy (Figure 3c), finer second-phase particles were observed, and EDS elemental mappings revealed overlapping enrichment of Sc and Zr in these particles, suggesting the formation of ternary intermetallic phases. Compared with the Sc-free alloy, the number density of intermetallic particles increased, while their average size decreased.

As the Sc content increased to 0.10 wt.% and 0.20 wt.% (Figure 3d,e), the intermetallic particles became more uniformly distributed and exhibited relatively small sizes. The corresponding EDS mappings showed a homogeneous distribution of Sc and Zr throughout the matrix, with pronounced co-enrichment in the intermetallic regions. These features indicate a progressive increase in the participation of Sc in intermetallic formation with increasing Sc content.

For the alloy containing 0.30 wt.% Sc (Figure 3f), a higher volume fraction of intermetallic particles was observed. Although ultrasonic treatment refined a portion of these particles, localized regions with relatively coarse intermetallics were still present. EDS mappings revealed strong co-segregation of Sc and Zr in these regions, consistent with the phase equilibrium analysis indicating the coexistence of multiple ternary phases at this Sc content.

### 3.3. Microstructural Evolution After Heat Treatment

Figure 4 shows SEM micrographs and corresponding EDS elemental mappings of the Al-0.3Zr-xSc alloys after 25 h heat treatment. Compared with the as-cast condition, the microstructures of all alloys exhibited a more homogeneous distribution of intermetallic particles after heat treatment, indicating the occurrence of precipitation and redistribution processes.

In the Sc-free Al-0.3Zr alloy, Zr-rich particles appeared more uniformly dispersed after heat treatment, suggesting partial homogenization and precipitation of Al_3_Zr from the aluminum matrix. For the Al-0.3Zr-0.05Sc alloy, the Sc and Zr containing intermetallic particles became finer and more evenly distributed, with reduced particle clustering compared with the as-cast state.

In alloys containing 0.10 wt.% and 0.20 wt.% Sc, the intermetallic particles after heat treatment were characterized by a high number density and small particle size, and the EDS mappings showed a uniform distribution of Sc and Zr throughout the matrix. These features indicate that heat treatment effectively promoted the formation of finely dispersed ternary precipitates in alloys with moderate Sc contents.

In contrast, the alloy with 0.30 wt.% Sc still exhibited localized regions with relatively higher intermetallic particle density after heat treatment. Although the overall distribution was more uniform than in the as-cast condition, particle coarsening in these regions remained evident, suggesting that excessive Sc addition promotes intermetallic aggregation even after prolonged heat treatment.

### 3.4. Grain Morphology and Grain Size Evolution

**Figure 5:**
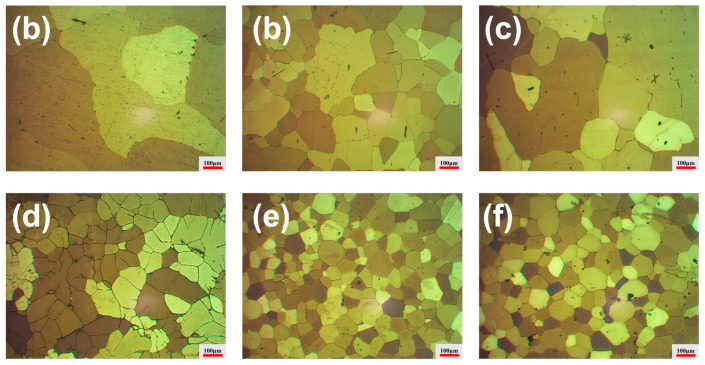
Low-magnification optical micrographs of Al–0.3Zr–xSc alloys after anodizing film treatment: (**a**) Al-0.3Zr (without ultrasonic treatment); (**b**) Al-0.3Zr; (**c**) Al-0.3Zr-0.05Sc; (**d**) Al-0.3Zr-0.1Sc; (**e**) Al-0.3Zr-0.2Sc; (**f**) Al-0.3Zr-0.3Sc.

**Figure 6:**
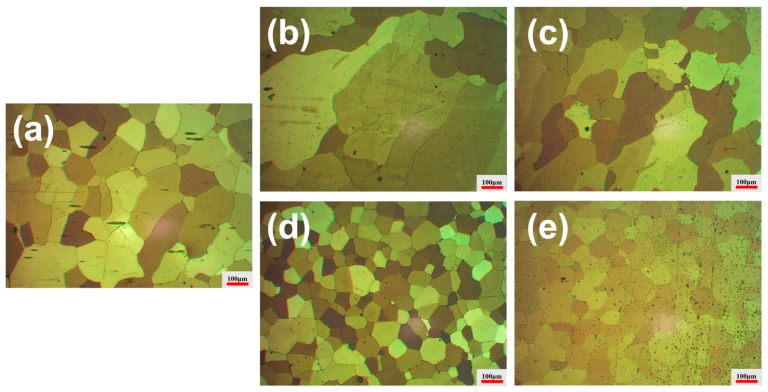
Low-magnification optical micrographs of Al–0.3Zr–xSc alloys after 25 h heat treatment (anodized): (**a**) Al-0.3Zr; (**b**) Al-0.3Zr-0.05Sc; (**c**) Al-0.3Zr-0.10Sc; (**d**) Al-0.3Zr-0.20Sc; (**e**) Al-0.3Zr-0.30Sc.

**Figure 7:**
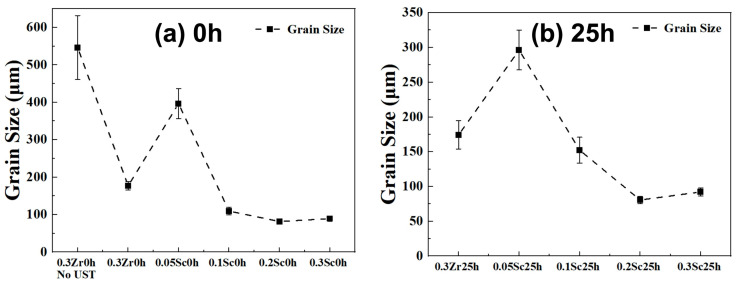
Grain size statistics of Al-0.3Zr-xSc alloys: (**a**) 0 h heat treatment (**b**) after 25 h heat treatment.

To evaluate the grain size and overall microstructural refinement, low-magnification optical micrographs of the anodized samples are presented in Figure 5 and Figure 6. Figure 5 presents the optical micrographs of the anodized samples in the as-cast condition, while the corresponding quantitative grain size statistics are summarized in Figure 7a. The Al–0.3Zr alloy without ultrasonic treatment exhibits coarse and non-uniform grains, with an average grain size of 546.2 ± 85.5 μm. After ultrasonic treatment, the Sc-free Al–0.3Zr alloy shows a pronounced grain refinement, and the average grain size is reduced to 177.0 ± 11.2 μm, corresponding to a reduction of nearly 68% compared with the non-ultrasonically treated alloy. This condition serves as the reference state for evaluating the effect of subsequent Sc addition. It is also worth noting that all quantitative values are reported as mean ± standard deviation, unless stated otherwise.

When 0.05 wt.% Sc is introduced, an unexpected grain coarsening phenomenon is observed. As shown in Figure 7a, the average grain size of the Al–0.3Zr–0.05Sc alloy increases markedly to 396.3 ± 40.2 μm, which is more than twice that of the Sc-free alloy subjected to ultrasonic treatment. This abnormal grain growth is also reflected in the less uniform grain morphology observed in Figure 5c.

With further increasing Sc content to 0.10 wt.% and 0.20 wt.%, a pronounced grain refinement is achieved. The average grain size decreases sharply to 109.7 ± 10.2 μm at 0.10 wt.% Sc and further to 81.7 ± 4.2 μm at 0.20 wt.% Sc, representing reductions of approximately 38% and 54%, respectively, compared with the Sc-free alloy under ultrasonic treatment. Among all investigated compositions, the alloy containing 0.20 wt.% Sc exhibits the finest and most homogeneous grain structure. When the Sc content reaches 0.30 wt.%, a slight grain coarsening tendency is observed, with an average grain size of 89.4 ± 6.6 μm; however, the grain size remains significantly smaller than that of the Sc-free alloy.

Figure 6 and Figure 7b show the grain structures and corresponding grain size evolution after 25 h heat treatment. The average grain size of the Sc-free Al–0.3Zr alloy remains nearly unchanged at 174.3 ± 20.3 μm, whereas the Al–0.3Zr–0.05Sc alloy still exhibits relatively coarse grains with an average size of 296.3 ± 28.4 μm. In contrast, alloys containing 0.10 wt.% and 0.20 wt.% Sc retain refined grain structures, with average grain sizes of 152.3 ± 18.6 μm and 80.6 ± 5.3 μm, respectively. The alloy with 0.30 wt.% Sc shows an average grain size of 92.2 ± 5.9 μm. These results quantitatively demonstrate that the grain refinement effect induced by Sc addition under ultrasonic treatment becomes effective only when the Sc content exceeds a critical threshold. It is also worth noting that no measurable porosity was detected in the metallographic observations of the as-cast alloys within the resolution of the present analysis. Although casting processes may potentially introduce shrinkage or gas porosity, no obvious pores or casting defects were observed in the examined samples. Therefore, porosity is not considered a governing factor influencing the mechanical or electrical performance in this study.

### 3.5. Electrical Conductivity Evolution

**Figure 8:**
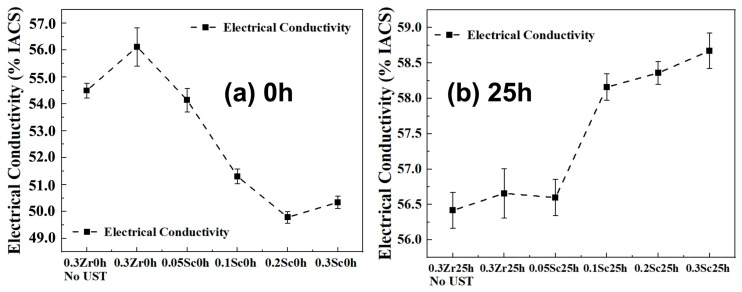
Variation in electrical conductivity (in % IACS) of Al-0.3Zr-xSc alloys as a function of alloy composition, measured at two different heat treatment conditions: (**a**) 0 h; (**b**) 25 h.

The variation in electrical conductivity of the Al–0.3Zr–xSc alloys as a function of Sc content is shown in Figure 8. In the as-cast condition (Figure 8a), the Sc-free Al–0.3Zr alloy without ultrasonic treatment exhibits an electrical conductivity of 54.49 ± 0.28% IACS, which increases to 56.12 ± 0.71% IACS after ultrasonic treatment. With the addition of Sc, the electrical conductivity decreases progressively to 54.14 ± 0.44% IACS at 0.05 wt.% Sc, 51.31 ± 0.27% IACS at 0.10 wt.% Sc, and 49.79 ± 0.22% IACS at 0.20 wt.% Sc, reflecting increased solute content and enhanced electron scattering. A slight recovery to 50.34 ± 0.23% IACS is observed at 0.30 wt.% Sc.

After 25 h of heat treatment (Figure 8b), the electrical conductivity of all alloys increases. The Sc-free alloy reaches 56.66 ± 0.35% IACS, while alloys containing 0.10 wt.% and 0.20 wt.% Sc exhibit conductivities of 58.16 ± 0.19% IACS and 58.36 ± 0.16% IACS, respectively. The alloy with 0.30 wt.% Sc shows a conductivity of 58.67 ± 0.25% IACS, indicating that moderate Sc addition allows effective conductivity recovery after heat treatment.

### 3.6. Tensile Properties

**Figure 9:**
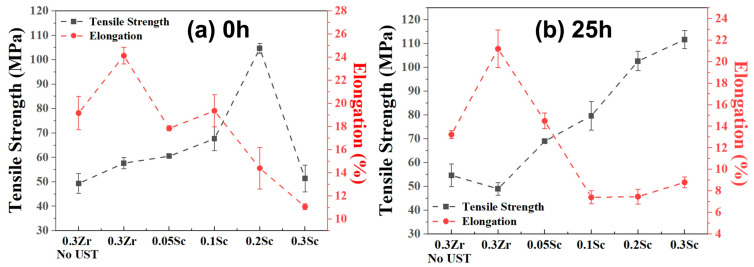
Tensile properties of Al-0.3Zr-xSc alloys: (**a**) 0 h heat treatment; (**b**) after 25 h heat treatment.

Figure 9 presents the tensile strength and elongation of the Al–0.3Zr–xSc alloys measured in the as-cast and heat-treated conditions. In the as-cast state (Figure 9a), the Sc-free Al–0.3Zr alloy without ultrasonic treatment exhibits a tensile strength of 49.3 ± 4.0 MPa and an elongation of 19.2 ± 1.4%. After ultrasonic treatment, the tensile strength increases to 57.7 ± 2.3 MPa, accompanied by an elongation of 24.1 ± 0.7%. With the addition of Sc, the tensile strength further increases to 60.5 ± 0.7 MPa at 0.05 wt.% Sc and 67.7 ± 4.9 MPa at 0.10 wt.% Sc, while elongation decreases moderately. A pronounced strength increase is observed at 0.20 wt.% Sc, reaching 104.7 ± 2.1 MPa, although elongation decreases to 14.4 ± 1.8%. In contrast, the alloy containing 0.30 wt.% Sc exhibits a reduced tensile strength of 51.3 ± 5.5 MPa and a low elongation of 11.1 ± 0.3%.

After 25 h of heat treatment (Figure 9b), tensile strength is further enhanced. The Sc-free alloy shows a tensile strength of 49.0 ± 2.6 MPa with an elongation of 21.2 ± 1.7%, while alloys containing 0.05 wt.% and 0.10 wt.% Sc exhibit tensile strengths of 69.0 ± 1.0 MPa and 79.7 ± 6.0 MPa, respectively. The highest tensile strength of 102.7 ± 4.0 MPa is achieved at 0.20 wt.% Sc, accompanied by an elongation of 7.5 ± 0.7%. Further increasing Sc content to 0.30 wt.% results in a tensile strength of 111.7 ± 3.8 MPa, with a limited elongation of 8.8 ± 0.5%, indicating a deterioration in ductility.

## 4. Discussion

### 4.1. Thermodynamic Background and Ultrasonic Interaction with Multiphase Melt

Figure 2 provides a thermodynamic context for interpreting the composition dependence of ultrasonic melt treatment (UST). At the UST temperature (710 °C), the investigated Al-0.3Zr-xSc melts are located in multiphase regions containing liquid Al together with primary Al_3_Zr and, depending on Sc level, additional ternary phases (τ_1_ or τ_2_). Therefore, UST acts on a particle-containing melt rather than a purely liquid melt, and the type/amount of intermetallic particles present during UST will influence the subsequent nucleation and solidification pathways [10].

UST introduces high-amplitude sound waves into the melt and is mainly governed by transient cavitation and acoustic streaming. Cavitation involves the formation and violent collapse of bubbles, producing localized high pressures/temperatures, shock waves and micro-jets, whereas acoustic streaming generates steady melt flow that enhances convection and solute transport [37,38].

When UST is applied to a multiphase melt, cavitation- and flow-induced stresses can directly modify the morphology, size and spatial distribution of solid intermetallic particles prior to final solidification. Because both cavitation activity and particle dynamics depend on melt constitution (e.g., particle volume fraction, viscosity and attenuation), the microstructural response to UST is expected to be strongly composition-dependent [39,40].

### 4.2. Cavitation and Acoustic Streaming-Induced Redistribution of Zr and Sc

The SEM/EDS results (Figure 3 and Figure 4) indicate that UST improves the spatial uniformity of Zr and Sc in both the as-cast and heat-treated states. This is consistent with the combined action of cavitation-driven particle fragmentation and acoustic streaming-enhanced melt convection, which can reduce solute concentration gradients and suppress macrosegregation [30,37].

In Al-Zr-containing melts, UST has been reported to refine and redistribute primary Al3Zr intermetallics, typically decreasing particle size while increasing particle number density and dispersion uniformity, although the details depend on the processing window (above/below liquidus) and the available undercooling [39,41,42]. Such particle conditioning is important because well-dispersed intermetallics and a more homogeneous solute field provide a more favorable starting condition for subsequent precipitation during heat treatment.

In addition to classical sonocrystallization or sonofragmentation arguments, our previous work showed that cavitation-induced heat generation and microjet-driven mass transfer can also contribute to the morphology evolution of Al_3_Zr particles under high-amplitude ultrasound in molten Al, promoting a transition from needle-like to thicker/rounded shapes as the vibration amplitude increases [32].

Extending these insights to the present Al-0.3Zr-xSc alloys, UST is expected to simultaneously (i) condition Al_3_Zr/ternary intermetallic particles and (ii) homogenize Zr/Sc partitioning in the melt, which helps explain the more uniform microstructures observed and the enhanced property response after a single 25 h heat treatment.

### 4.3. Sc-Content-Dependent Transition of Dominant Microstructural Control Mechanisms

The grain-size response to Sc addition under ultrasonic treatment (UST) is clearly non-monotonic. Without UST, the Sc-free alloy exhibits very coarse grains (546.2 ± 85.5 μm), whereas UST refines it to 177.0 ± 11.2 μm. With Sc addition, an unexpected coarsening occurs at 0.05 wt.% Sc (396.3 ± 40.2 μm), followed by a sharp refinement at 0.10 wt.% Sc (109.7 ± 10.2 μm) and an optimum at 0.20 wt.% Sc (81.7 ± 4.2 μm). A further increase to 0.30 wt.% Sc results in only a slight coarsening (89.4 ± 6.6 μm). After 25 h heat treatment, the grain-size hierarchy is largely preserved (80.6 ± 5.3 μm for 0.20 wt.% Sc), indicating that the solidification-established grain structure is not erased by subsequent thermal exposure.

#### 4.3.1. Low-Sc Regime (≤0.05 wt.%): Reduced Population of Effective Nuclei

Under the present processing route (UST at 710 °C), the phase constitution during ultrasound exposure is expected to vary with Sc content, potentially altering both the type and fraction of solid intermetallic particles present in the melt. UST performed above the aluminum liquidus is reported to be most effective when applied within the temperature range where primary Al_3_Zr can exist, because cavitation may enhance nucleation on oxides and fragment/refine Al_3_Zr crystals, thereby increasing the number of potential substrates for α-Al nucleation [40]. High-amplitude ultrasound has also been shown to modify the morphology of Al_3_Zr particles in molten aluminum via cavitation-related heat and mass transfer effect [32]. In addition, crystallographic analyses suggest that Al_3_Zr can form low-misfit orientation relationships with α-Al, which may enable Al_3_Zr to act as a nucleation substrate when sufficiently fine and well dispersed [43].

In this context, the pronounced grain coarsening at 0.05 wt.% Sc (396.3 ± 40.2 μm) may indicate that the population of effective nucleation sites is lower than in the UST-treated Sc-free alloy. One possible explanation is that introducing a small amount of Sc modifies the balance among intermetallic substrates in the multiphase melt, e.g., by promoting the formation of Sc–Zr–Al ternary intermetallic(s) (denoted as τ-type phases in the thermodynamic analysis) that may (i) consume part of Zr/Sc from the liquid and/or (ii) influence the size/number density and nucleation potency of available substrates. Because nucleation potency is influenced by crystallographic matching and interfacial energy, different intermetallic species may not contribute equally to grain refinement.

It should be noted that this interpretation is primarily based on qualitative microstructural observations and established precipitation theory. In the absence of quantitative particle number density measurements, size distribution analysis, and phase identification by EBSD or TEM, the proposed mechanism should be regarded as a working hypothesis rather than a confirmed explanation. Further quantitative characterization would be required to distinguish between “substrate scarcity” and “substrate potency” effects in this low-Sc regime.

The contribution of grain refinement to strength can be described by the classical Hall–Petch relationship (3) [44],

(3) $$\sigma_{y}=\sigma_{0}+k_{y}d^{-1/2}$$

where σ_y_ is the yield strength, σ_0_ is the lattice friction stress, k_y_ is the Hall–Petch coefficient, and d is the average grain size. Therefore, the comparatively coarse grains at 0.05 wt.% Sc are expected to provide only a more limited grain-size strengthening contribution compared with the more refined alloys.

#### 4.3.2. Intermediate-Sc Regime (0.10–0.20 wt.%): Activated Synergistic Refinement

When the Sc content increases to 0.10–0.20 wt.%, the grain size drops sharply (109.7 ± 10.2 μm and 81.7 ± 4.2 μm). In this composition range, the thermodynamically predicted melt state at 710 °C tends to shift toward an L + τ intermetallic field. Under such conditions, UST can act in two complementary ways: (i) cavitation-induced fragmentation and de-agglomeration increase the number density of small, well-dispersed intermetallic particles that can act as heterogeneous nuclei, and (ii) acoustic streaming homogenizes temperature/solute fields, reducing macrosegregation and stabilizing an equiaxed growth front [41]. From the broader viewpoint of grain refinement theory, effective refinement requires both potent nuclei and sufficient solute-based growth restriction; thus, increasing Sc can activate refinement by simultaneously modifying the nucleant population and increasing growth restriction at the solid–liquid interface.

The retention of a fine grain size after 25 h heat treatment (e.g., 80.6 ± 5.3 μm for 0.20 wt.% Sc) further suggests strong resistance to grain growth in this regime. In Al–Sc–Zr systems, coherent L1_2_ Al_3_(Sc, Zr) precipitates/dispersoids with a Sc-rich core and a Zr-rich shell have been widely reported; these precipitates exhibit enhanced coarsening resistance and can effectively pin dislocations and grain/subgrain boundaries, contributing to microstructural stability during thermal exposure [10].

#### 4.3.3. High-Sc Regime (0.30 wt.%): Saturation of Refinement Mechanisms

Increasing Sc to 0.30 wt.% does not lead to further grain refinement; instead, a slight coarsening to 89.4 ± 6.6 μm is observed. At higher Sc levels, the equilibrium melt state at 710 °C contains a higher fraction of solid τ-type intermetallic phases, which can introduce competing effects: while a larger intermetallic population could in principle provide more nucleation substrates, a higher particle loading also increases the likelihood of particle agglomeration/sedimentation and can attenuate or redistribute the ultrasonic field, making the cavitation zone more localized. Such saturation of refinement efficiency with increasing solid-particle content is consistent with observations from Eskin et al. [30].

Overall, the present results indicate a composition-dependent transition of the dominant grain-structure control mechanism: a low-Sc regime where the effective nucleant population is reduced (0.05 wt.% Sc), an intermediate regime where UST and Sc jointly activate strong refinement (0.10–0.20 wt.% Sc), and a high-Sc regime where refinement saturates due to competing multiphase effects (0.30 wt.% Sc). This provides a mechanistic basis for selecting a narrow Sc window (~0.10–0.20 wt.%) to maximize grain refinement and subsequent property benefits under the current UST conditions.

### 4.4. Precipitation Kinetics and Implications for Aging Efficiency

The strengthening and conductivity response of Al-Zr-Sc alloys is generally attributed in the literature to the precipitation of Zr- and Sc-containing L_12_ dispersoids. In Zr-bearing Al alloys, precipitation strengthening is often sluggish because Zr has very low diffusivity in Al, and heat treatments are typically designed to promote fine L_12_-Al_3_Zr dispersoids while avoiding transformation to non-coherent D_023_-Al_3_Zr at higher temperatures [45]. In Al-Sc-Zr alloys, Sc-rich L_12_ precipitates have been reported to form first and subsequently develop Zr-enriched shells, which is associated with improved coarsening resistance and thermal stability [46].

For diffusion-controlled precipitation processes, the characteristic diffusion distance *x* can be approximated as Formula (4) [47]:

(4) $$x\propto \sqrt{\mathrm{Dt}}$$

where *D* is the diffusion coefficient and *t* is the aging time. UST may reduce microsegregation before solidification and condition intermetallic particles (smaller size, higher dispersion), which may in turn reduce the local diffusion distances required for solute redistribution and precipitation during subsequent heat treatment.

Although a fully time-dependent ageing study is still required to quantitatively evaluate possible kinetic acceleration, the present data demonstrate that a single 25 h heat treatment yields a substantial conductivity recovery (up to 58.67% IACS) and significant strengthening in Sc-containing alloys (up to 111.7 MPa). These results indicate that UST-assisted solute homogenization combined with Sc microalloying is compatible with achieving a favorable strength–conductivity balance under the applied aging condition. However, without a systematic comparison of conductivity evolution and time-to-plateau behavior between UST-treated and non-UST alloys, the present findings should be interpreted as suggesting a potential for improved aging efficiency rather than conclusively demonstrating a reduction in required heat treatment duration [11].

It also should be emphasized that direct nanoscale characterization of Zr- and Sc-containing L_12_ precipitates was not performed in the present study. Therefore, the discussion regarding precipitate structure, core–shell morphology, and diffusion-controlled growth behavior is based on established literature and indirect experimental indicators (e.g., conductivity evolution and mechanical response), rather than direct microstructural confirmation. Future work involving TEM analysis will be necessary to quantitatively determine precipitate size distribution, number density, and phase constitution.

### 4.5. Electrical Conductivity: Solute State and Scattering Mechanisms

Electrical conductivity evolution in the Al–0.3Zr–xSc alloys can be quantitatively interpreted using Matthiessen’s rule (5), which describes the total electrical resistivity as the sum of various scattering contributions [48]:

(5) $$\rho =\rho_{0}+\sum \rho_{i}$$

where ρ is the total electrical resistivity, ρ_0_ is the intrinsic resistivity of pure aluminum at the measurement temperature, and ρ_i_ represents resistivity contributions from solute atoms, lattice defects and microstructural features (e.g., precipitates, grain boundaries) [49].

In the as-cast condition, adding Sc (0.05–0.20 wt.%) decreases conductivity from 56.12% IACS (Sc-free, with UST) to 52.72% IACS (0.20 wt.% Sc), consistent with increased solid-solution scattering and the presence of Sc/Zr-containing intermetallics. After 25 h heat treatment, conductivity increases for all alloys (up to 58.67% IACS at 0.30 wt.% Sc), which is consistent with solute depletion from the matrix via precipitation and with recovery of quenched-in defects [46,50].

Notably, the heat-treated alloys containing 0.10–0.30 wt.% Sc reach 58.16–58.67% IACS, which is comparable to reported values for thermal-resistant Al-Zr-Sc conductor alloys (typically ~57–60% IACS) while offering increased strength [7,11]. From a balance perspective, 0.10–0.20 wt.% Sc provides a substantial conductivity recovery together with significant grain refinement and strengthening, whereas higher Sc levels may increase the risk of forming coarse intermetallic clusters that can compromise ductility (Section 4.3.3).

Overall, conductivity recovery after heat treatment is dominated by the solute state (in-solution vs precipitated), highlighting the importance of UST-assisted solute homogenization and dispersoid formation for conductor-related applications.

### 4.6. Strength-Ductility Balance from a Microstructural Perspective

The tensile properties result from the combined effects of grain structure (Hall-Petch strengthening), solid-solution/precipitation strengthening and the damage tolerance of the microstructure. In the as-cast state, the 0.20 wt.% Sc alloy exhibits both the finest grains (81.7 ± 23.0 μm) and the highest tensile strength (104.7 ± 3.0 MPa), consistent with a synergistic contribution from grain refinement and a higher density of strengthening phases/dispersoids promoted by Sc additions [45]. After heat treatment, the Sc-free alloy shows a reduced tensile strength (49.0 ± 2.6 MPa), which is consistent with recovery/annealing dominating over Zr precipitation strengthening when diffusion is sluggish [12]. In contrast, Sc-containing alloys retain much higher strengths (79.7–111.7 MPa), consistent with precipitation/dispersoid strengthening in Al-Sc-Zr systems [51].

Ductility decreases significantly once Sc reaches 0.20 wt.% (elongation ~7.5–8.8% after heat treatment), which can be rationalized by the increased tendency to form coarse intermetallic particles/clusters that act as crack initiation sites and reduce the uniform plastic strain [52]. It should be noted that fracture surface analysis was not performed in the present study. Therefore, the proposed interpretation regarding crack initiation at coarse intermetallic particles or clusters remains a plausible microstructural explanation based on SEM observations rather than a directly verified fracture mechanism. Coarse or clustered τ-type intermetallic phases, if present, may promote localized stress concentration and facilitate crack propagation under tensile loading. Future fractographic characterization using SEM would be required to confirm the dominant failure mode and clarify the relationship between intermetallic distribution and ductility loss at higher Sc contents. This highlights that strength improvements obtained by increasing the volume fraction of strengthening phases must be balanced against the accompanying loss of damage tolerance.

Overall, the present data suggest that intermediate Sc additions (0.10–0.20 wt.% Sc) provide a practical strength–conductivity balance after a single 25 h heat treatment, whereas higher Sc (0.30 wt.% Sc) yields the highest strength and conductivity but requires careful control of intermetallic particle clustering to preserve ductility.

### 4.7. Advancement Beyond Prior Work

Compared with previous studies, the present work advances the understanding of ultrasonic-assisted processing of heat-resistant aluminum alloys by integrating a well-established mechanistic framework for ultrasonic modification of Al_3_Zr intermetallics with Sc microalloying. While prior work clarified the physical mechanisms governing ultrasonic-Al_3_Zr interactions, the present study demonstrates how composition-dependent Sc effects activate or suppress microstructural control mechanisms under ultrasonic treatment.

This combined process–chemistry perspective provides new insight into threshold-dependent microstructural evolution and offers a more efficient strategy for balancing strength and electrical conductivity.

### 4.8. Industrial Implications: Potential of Heat Treatment Time Reduction

From an industrial perspective, the most significant implication of this study lies in the feasibility of reducing heat treatment time through ultrasonic-assisted Sc microalloying. Alloys containing 0.10–0.20 wt.% Sc exhibit refined and thermally stable microstructures, favorable electrical conductivity, and enhanced mechanical properties after relatively short heat treatment durations.

This strategy provides a practical pathway for improving production efficiency and reducing energy consumption in the manufacture of heat-resistant aluminum conductor alloys.

It should be emphasized that the mechanical properties reported in the present study correspond to the as-cast condition, with the primary objective of clarifying the fundamental effects of ultrasonic treatment on solidification behavior and precipitation evolution. In contrast, commercial high-strength aluminum alloys such as AA2024 and AA7075 are typically evaluated in wrought and peak-aged tempers, where thermomechanical processing plays a dominant role in strengthening. Therefore, direct quantitative comparison with these alloys at the current stage is limited. A systematic evaluation after subsequent rolling and optimized aging treatments will be conducted in future work to further assess the engineering competitiveness of the present alloy system.

## 5. Conclusions

The combined effects of Sc microalloying and ultrasonic treatment (UST) on Al–0.3Zr–xSc alloys (x = 0–0.30 wt.%) were clarified. The main conclusions are as follows:

1. Thermodynamic phase-equilibrium analysis indicates that increasing Sc changes the solid–liquid coexistence state at 710 °C, resulting in different intermetallic phase populations present during UST and thus different microstructural conditioning responses.
2. UST refines and homogenizes Zr/Sc-containing intermetallic compounds, improving the distribution uniformity of Zr and Sc in the as-cast alloys and providing a potentially more favorable starting state for subsequent precipitation during aging.
3. Grain refinement exhibits a pronounced threshold behavior with Sc content: relative to the UST-treated Sc-free alloy (~177 μm), 0.05 wt.% Sc shows abnormal coarsening (~396 μm), whereas 0.10–0.20 wt.% Sc produces strong refinement (~110 to ~82 μm), and the refined grain structures are largely retained after 25 h of aging.
4. Electrical conductivity decreases with increasing Sc in the as-cast condition but recovers after aging; alloys with moderate Sc contents reach about 58% IACS after 25 h.
5. A favorable strength–conductivity balance is achieved at moderate Sc contents. The 0.20 wt.% Sc alloy attains >100 MPa tensile strength together with approximately 58% IACS after 25 h of aging, while a higher Sc content further increases strength but with reduced ductility.

These results indicate that optimizing Sc content (0.10–0.20 wt.%) in combination with UST represents a composition-dependent strategy to tailor grain structure and intermetallic distribution and to improve the comprehensive performance of heat-resistant Al–Zr conductor alloys. Importantly, achieving this balance after 25 h of aging suggests that ultrasonic-assisted Sc microalloying is compatible with improved precipitation response under the present aging condition. However, a systematic time-dependent comparison between UST-treated and non-UST alloys is required to quantitatively assess any reduction in required aging duration.

## Figures and Tables

**Table 1:** The nominal compositions and measured elemental contents of the alloys.

Alloy Composition	Zr (wt.%)		Sc (wt.%)	
	Target	Measured	Target	Measured
Al-0.3Zr	0.30	0.19	-	-
Al-0.3Zr-0.05Sc	0.30	0.24	0.05	0.04
Al-0.3Zr-0.1Sc	0.30	0.33	0.10	0.12
Al-0.3Zr-0.2Sc	0.30	0.17	0.20	0.22
Al-0.3Zr-0.3Sc	0.30	0.19	0.30	0.28

## Data Availability

The original contributions presented in this study are included in the article. Further inquiries can be directed to the corresponding author.
